# Functional Complexes of Angiotensin-Converting Enzyme 2 and Renin-Angiotensin System Receptors: Expression in Adult but Not Fetal Lung Tissue

**DOI:** 10.3390/ijms21249602

**Published:** 2020-12-16

**Authors:** Rafael Franco, Alejandro Lillo, Rafael Rivas-Santisteban, Ana I. Rodríguez-Pérez, Irene Reyes-Resina, José L. Labandeira-García, Gemma Navarro

**Affiliations:** 1 Laboratory of Molecular Neurobiology, Department Biochemistry and Molecular Biomedicine, School of Biology, University of Barcelona, 08007 Barcelona, Spain; alilloma55@gmail.com (A.L.); rrivasbioq@gmail.com (R.R.-S.); ire-reyes@hotmail.com (I.R.-R.); 2 Network Center, Neurodegenerative Diseases (CiberNed), Spanish National Health Institute Carlos III, Valderrebollo 5, 28031 Madrid, Spain; anai.rodriguez@usc.e (A.I.R.-P.); joseluis.labandeira@usc.es (J.L.L.-G.); 3 Laboratory of Cellular and Molecular Neurobiology of Parkinson’s Disease, Research Center for Molecular Medicine and Chronic Diseases (CIMUS), Department of Morphological Sciences, IDIS, University of Santiago de Compostela, 15705 Santiago de Compostela, Spain; 4 RG Neuroplasticity, Leibniz Institute for Neurobiology, 39118 Magdeburg, Germany; 5 Department of Biochemistry and Physiology, School of Pharmacy and Food Science, University of Barcelona, 08007 Barcelona, Spain

**Keywords:** COVID-19, SARS-CoV-2 receptor, RAS, ACE2, angiotensin receptor, Mas receptor, lung

## Abstract

Angiotensin-converting enzyme 2 (ACE2) is a membrane peptidase and a component of the renin-angiotensin system (RAS) that has been found in cells of all organs, including the lungs. While ACE2 has been identified as the receptor for severe acute respiratory syndrome (SARS) coronaviruses, the mechanism underlying cell entry remains unknown. Human immunodeficiency virus infects target cells via CXC chemokine receptor 4 (CXCR4)-mediated endocytosis. Furthermore, CXCR4 interacts with dipeptidyl peptidase-4 (CD26/DPPIV), an enzyme that cleaves CXCL12/SDF-1, which is the chemokine that activates this receptor. By analogy, we hypothesized that ACE2 might also be capable of interactions with RAS-associated G-protein coupled receptors. Using resonance energy transfer and cAMP and mitogen-activated protein kinase signaling assays, we found that human ACE2 interacts with RAS-related receptors, namely the angiotensin II type 1 receptor (AT_1_R), the angiotensin II type 2 receptor (AT_2_R), and the MAS1 oncogene receptor (MasR). Although these interactions led to various alterations of signal transduction, but, more importantly, ligand binding to AT_1_R resulted in the downregulation of ACE2 cell surface expression, while ligand binding to AT_2_R, but not to MasR, resulted in upregulation of ACE2 cell surface expression. Proximity ligation assays performed in situ revealed macromolecular complexes containing ACE2 and AT_1_R, AT_2_R or MasR in adult but not fetal mouse lung tissue. These findings highlight the relevance of RAS in SARS-CoV-2 infection and the role of ACE2-containing complexes as potential therapeutic targets.

## 1. Introduction

The current coronavirus disease-2019 (COVID-19) pandemic is the result of widespread infection with the severe acute respiratory syndrome-coronavirus-2 (SARS-CoV-2) pathogen. The main cell surface receptor for SARS-CoV-2 is angiotensin-converting enzyme 2 (ACE2), which is an enzyme that catalyzes the conversion of angiotensin II (Ang II) into angiotensin 1-7 (Ang 1-7). The link between ACE2 and SARS coronaviruses was discovered serendipitously [1,2,3,4,5]. ACE2 is a component of the renin-angiotensin system (RAS), which has been characterized extensively in the kidney and serves as the target of efficacious antihypertensive drugs. In addition to enzymes that process renin and angiotensin, components of the RAS include members of the G-protein-coupled receptor (GPCR) superfamily. Receptors for Ang II type 1 (AT_1_Rs) and Ang II type 2 (AT_2_Rs) share Ang II as an endogenous ligand. As noted, Ang II is also a substrate for enzymatic processing by ACE2 [6]. By contrast, the Mas1 oncogene receptor (MasR) interacts with Ang 1-7. Mas1, also known as the Mas-related proto-oncogene, is related to a putative ancestor gene identified in *Saccharomyces cerevisiae* that encodes mitochondrial assembly protein-1 [7]. Additional RAS receptors, the Mas-related GPCRs (Mrgprs), are also responsive to Ang 1-7 [8,9,10] and to another endogenous agonist that is an Ang 1-7 derivative, alamandine [6].

Coronaviruses and the human immunodeficiency virus (HIV), the latter pathogen recognized as the causative agent of acquired immunodeficiency syndrome (AIDS), have several common characteristics. Both RNA viruses contain nucleic acids enveloped within a membrane that contains host components and viral proteins that facilitate interactions with surface receptors on target cells. The most studied of the HIV subtypes, HIV-1, interacts with target cell surface receptors and co-receptors that are critical for entry into the host cell. HIV-1 entry requires interactions with the main receptor, CD4, and interactions with a GPCR co-receptor, most notably the CXC chemokine receptor, CXCR4 [11,12,13,14,15,16,17]. The chemokine CXCL12, also known as stromal-derived factor 1 (SDF-1), is the endogenous ligand of CXCR4. Interestingly, CXCL12/SDF-1 is degraded by dipeptidyl peptidase-4 (CD26/DPPIV). The actions of this enzyme serve to reduce the local concentration of CXCL12/SDF-1 and thereby protect the host cells from viral infection [18,19]. ACE2 and CD26/DPPIV are both proteases with several specific structural similarities. For example, both ACE2 and CD26/DPPIV are attached to cell membranes and can be removed and released into body fluids via a process known as shedding [20,21]. Furthermore, both enzymes are type I transmembrane proteins with a single transmembrane domain, a C-terminal domain facing the cytoplasm, and a large N-terminal extracellular domain that includes the catalytic site.

Glycoprotein 120 kDa (gp120) found on the surface of HIV-1 virions interacts with CD26/DPPIV, which may interact with CXCR4. Among the findings that support our hypothesis, we previously characterized co-modulation of CXCR4 and CD26/DPPIV in human lymphocytes. We also found that the non-catalytic activating function of CD26/DPPIV was altered in the presence of gp120 via a mechanism that was dependent on the expression of both CD4 and CXCR4 [19,22].

Based on these findings, we hypothesized that the ACE2 may have the capacity to interact with receptors that are activated by its substrate, Ang II, and its product, Ang 1-7. Accordingly, this paper aimed at examining the physical and functional interactions of ACE2 with cell surface receptors for Ang II and Ang 1-7. We also performed experiments designed to detect enzyme-receptor complexes in lung tissue, which is the main portal of entry for SARS-CoV-2.

## 2. Results

### 2.1. Expression of ACE2 Downregulates AT_1_R-Mediated Signaling Induced by Ang II

**Figure 1:**
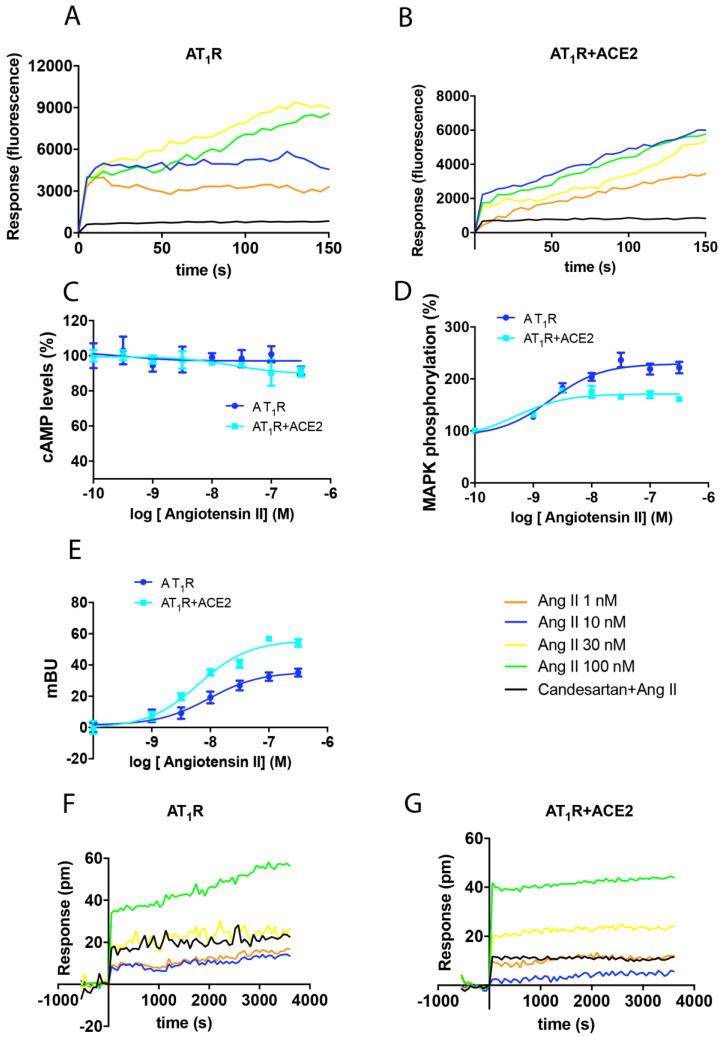
Impact of ACE2 on the functionality of AT_1_R. HEK-293T cells were transfected with either 0.4 µg AT_1_R cDNA and 0.2 µg ACE2-HA cDNA (**A**,**B**,**F**,**G**), 0.5 µg AT_1_R-YFP cDNA, 0.2 µg ACE2-HA cDNA, and 0.5 µg β-arrestin II-RLuc cDNA (**C**), or 0.4 µg AT_1_R cDNA, 0.2 µg ACE2-HA cDNA, and 0.5 µg cDNA encoding the Ca^2+^ sensor, GCaMP6 (**D**,**E**). After 48 h of incubation, cells were treated with increasing concentrations of the AT_1_R agonist, Ang II. Cyclic AMP was measured after 15 min in response to pre-treatment with 0.5 μM forskolin (**A**); as shown, this intervention resulted in approximately 4 nM cAMP, which corresponds to a 240% increase over baseline levels. Results from the evaluation of ERK1/2 phosphorylation (**B**), β-arrestin II recruitment (**C**) Ca^2+^ levels (**D**,**E**), and DMR recordings (**F**,**G**) are presented as dose-response curves. Where indicated, cells were pre-treated with the selective AT_1_R receptor antagonist, candesartan (1 µM), before challenge with the receptor agonist. Values shown are the mean ± SEM of 8 independent experiments each performed in triplicate.

ACE2 has been identified as the main receptor for SARS coronaviruses. Its substrate, Ang II, is an endogenous agonist that activates the G-protein-coupled receptors (GPCRs) AT_1_R and AT_2_R. AT_1_R couples with the Gq protein; thus, activation by agonists increases the levels of inositol triphosphate and diacylglycerol and mobilizes intracellular calcium. In this first set of experiments, we aimed to determine whether the expression of ACE2 had any impact on AT_1_R-mediated signaling. Toward this end, we measured cytoplasmic Ca^2+^ levels in a heterologous expression system using the calmodulin-derived Ca^2+^ sensor, GCaMP6. Ang II at concentrations of 1 nM to 100 nM was added to HEK-293T cells that expressed both AT_1_R and GCaMP6. A fluorescent signal with a maximum of 9000 AU at 150 s was detected in response to the two highest concentrations of Ang II (Figure 1A). Interestingly, in a similar experiment targeting HEK-293T cells expressing AT_1_R and ACE2, a significant decrease in the maximum response was observed (6000 AU signal at the highest concentration of Ang II; Figure 1B). These results suggest that the expression of ACE2 may inhibit AT_1_R-mediated signaling. The possibility of functional selectivity and Gi coupling was discarded from experiments that evaluated intracellular cAMP levels in the presence or absence of forskolin. No effect on cAMP levels was identified in response to micromolar concentrations of Ang II. Moreover, the expression of ACE2 had no significant impact on these results (Figure 1C).

Activation of GPCRs results in the engagement of the mitogen-activated protein kinase (MAPK) signaling pathway. As such, we measured extracellular signal-regulated kinase (ERK)1/2 phosphorylation in cells expressing AT_1_R and ACE2. While the addition of Ang II to HEK-293T cells expressing AT_1_R induced a 125% increase in ERK1/2 phosphorylation over baseline levels, the increase in phosphorylation observed in cells co-expressing AT_1_R and ACE2 was limited to 71% (Figure 1D). Similar results were obtained using dynamic mass redistribution (DMR) assays, which is a technique that can be used to measure changes in cytoskeletal structure in response to GPCR activation and the engagement of G-proteins. With this assay, we found that the expression of ACE2 resulted in a 30% decrease in the overall impact of Ang II at AT_1_R (Figure 1F,G). Finally, AT_1_R-mediated recruitment of β-arrestin was evaluated in cells that co-express β-arrestin II-RLuc and AT_1_R-YFP. The specific signal of 37 milli-Bioluminescence Resonance Energy Transfer (BRET) units (mBU) originally detected in cells expressing AT_1_R alone increased by 20 mBU in response to ACE2 co-expression (Figure 1E). These results suggest that the expression of ACE2 augments AT_1_R-mediated recruitment of β-arrestins.

Taken together, our findings revealed that the expression of ACE2 results in a decrease in AT_1_R-mediated signaling in response to Ang II with a concomitant increase in the capacity for β-arrestin recruitment.

### 2.2. Expression of ACE2 Downregulates AT_2_R-Mediated Signaling Induced by Ang II

**Figure 2:**
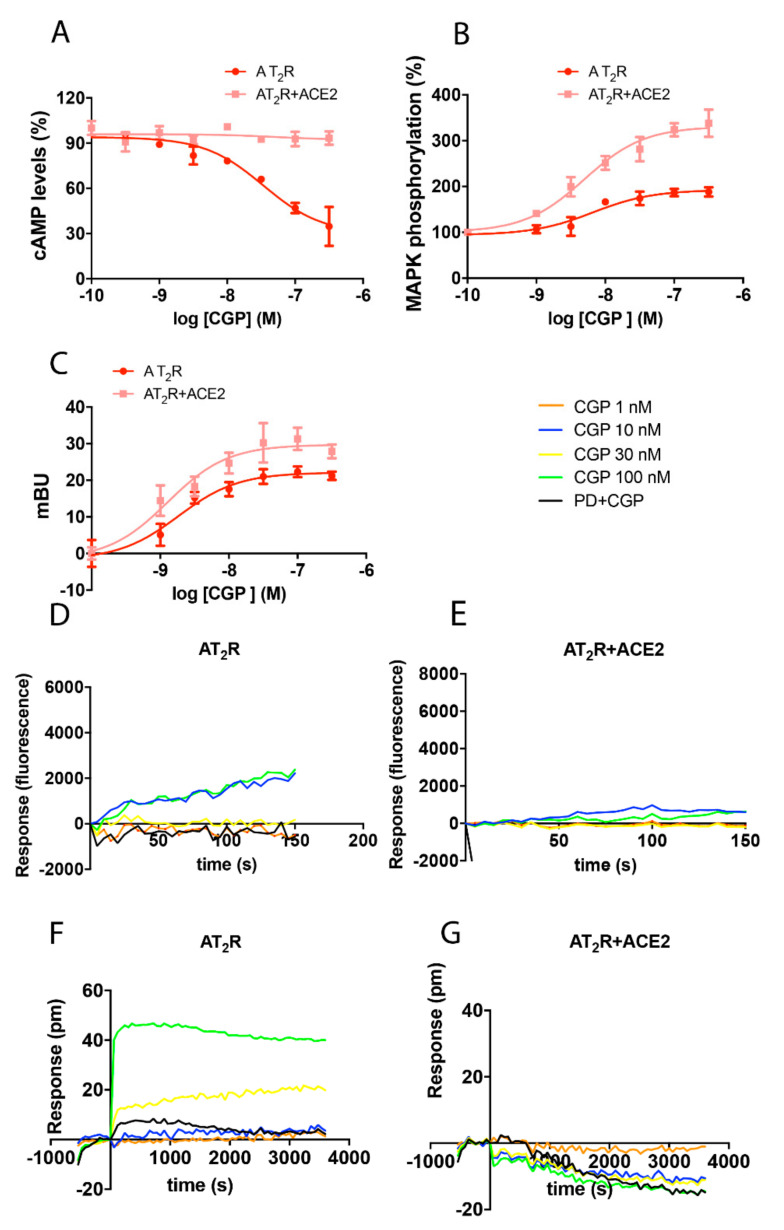
Impact of ACE2 on the functionality of AT_2_R. HEK-293T cells were transfected with either 0.3 µg AT_2_R cDNA and 0.2 µg ACE2-HA cDNA (**A**,**B**,**F**,**G**), 0.4 µg AT_2_R-YFP cDNA, 0.2 µg ACE2-HA cDNA, and 0.5 µg β-arrestin II-RLuc cDNA (**C**), or 0.3 µg AT_2_R cDNA, 0.2 µg ACE2-HA cDNA, and 0.5 µg cDNA encoding the Ca^2+^sensor, GCaMP6 (**D**,**E**). After 48 h of incubation, cells were treated with increasing concentrations of the selective AT_2_R agonist, CGP. Cyclic AMP was measured after 15 min in response to pre-treatment with 0.5 μM forskolin (**A**); see also Legend to Figure 1. Results from the evaluation of ERK1/2 phosphorylation (**B**), β-arrestin II recruitment (**C**), Ca^2+^ levels (**D**,**E**), and DMR recordings (**F**,**G**) are presented as dose-response curves. Where indicated, cells were pre-treated with selective AT_2_R receptor antagonist, PD123319 (PD; 1 µM), before challenge with the receptor agonist. Values are the mean ± SEM of 8 independent experiments each performed in triplicate.

Signaling assays were performed to assess the impact of ACE2 on AT_2_R function. As AT_2_R couples with Gi, we first determined intracellular cAMP levels in cells treated with forskolin. Findings shown in Figure 2A reveal that the addition of the selective AT_2_R agonist, CGP-42112A (CGP), resulted in a 73% reduction in cAMP levels. This strong effect was markedly attenuated in cells that co-expressed ACE2 (only 12% reduction in cAMP levels). While similar results were obtained in DMR assays (Figure 2F,G), our findings revealed qualitative differences with respect to the engagement of the MAPK signaling pathway. Of note, we found that ERK1/2 phosphorylation following activation of AT_2_R was increased from 90% to 232% in response to ACE2 (Figure 2B). These results indicate that ACE2 expression potentiates the link between AT_2_R and MAPK signaling.

By contrast, expression of ACE2 had a less profound impact on β-arrestin recruitment via AT_2_R when compared to responses mediated by AT_1_R. BRET_max_ was 22 mBU in cells expressing AT_2_R; this response increased to 29 mBU when ACE2 was also expressed (Figure 2C). As anticipated from findings of AT_2_R coupling with Gi and not to Gq, Ang II-mediated activation of this receptor-induced minimal mobilization of intracellular Ca^2+^. This response was completely abolished in the presence of ACE2 (Figure 2D,E).

### 2.3. Expression of ACE2 Potentiates MasR-Mediated Signaling

**Figure 3:**
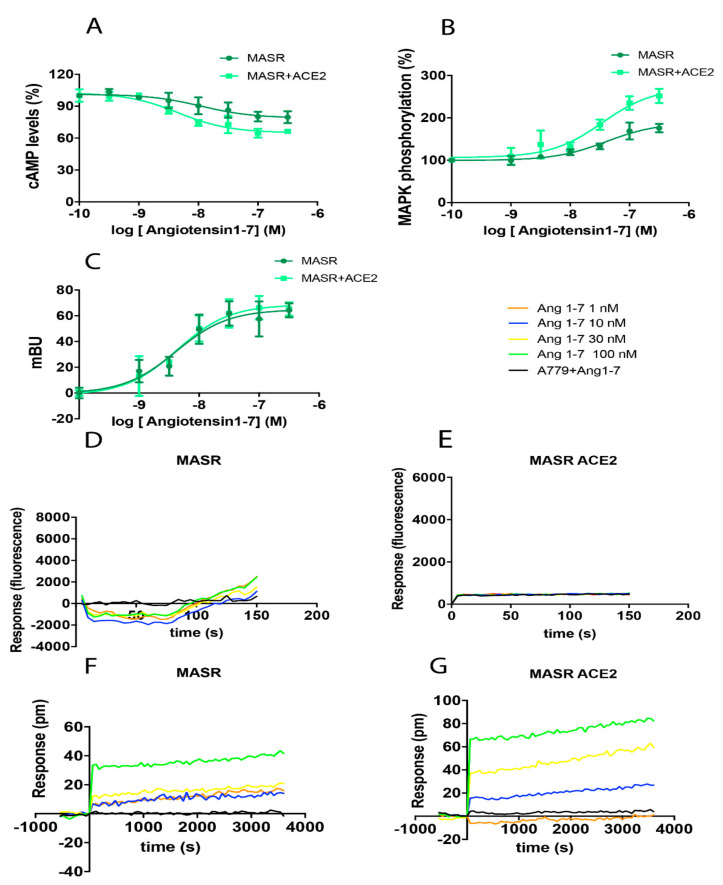
Impact of ACE2 on the functionality of MasR. HEK-293T cells were transfected with 0.5 µg MasR cDNA and 0.2 µg ACE2-HA cDNA (**A**,**B**,**F**,**G**), 0.6 µg MasR-YFP cDNA, 0.2 µg ACE2-HA cDNA, and 0.5 µg encoding β-arrestin II-RLuc cDNA (**C**), or 0.5 µg MasR cDNA, 0.2 µg ACE2-HA cDNA, and 0.5 µg cDNA encoding the Ca^2+^ sensor, GCaMP6 (**D**,**E**). After 48 h of incubation, cells were treated with increasing concentrations of the selective MasR agonist, Ang 1-7. Cyclic AMP was measured after 15 min in response to pre-treatment with 0.5 μM forskolin (**A**); see also Legend to Figure 1. Results from the evaluation of ERK1/2 phosphorylation (B), β-arrestin II recruitment (**C**), Ca^2+^ levels (**D**,**E**), and DMR recordings (**F**,**G**) are presented as dose-response curves. Where indicated, cells were pre-treated with the selective MasR receptor antagonist, A779 (1 µM), before challenge with the receptor agonist. Values presented are the mean ± SEM of 6 independent experiments each performed in triplicate.

Assays analogous to those described in earlier sections were performed to determine the impact of ACE2 on the functionality of MasR. While the recruitment of β-arrestin in response to receptor activation with its ligand, Ang 1-7, was not affected by co-expression of ACE2, expression of ACE2 resulted in the enhancement of all other signals transduced via this receptor. This was observed in assays targeting cAMP and ERK1/2 phosphorylation and was notably strong in DMR readouts (Figure 3). As such, not only does the catalytic activity of ACE2 generate the endogenous agonist for this receptor, our results reveal that the expression of this enzyme may increase the signaling output from MasR via an enzyme-independent mechanism. We confirmed that MasR activation resulted in no changes in cytoplasmic Ca^2+^ levels, regardless of the presence or absence of ACE2 (Figure 3D,E).

### 2.4. ACE2 Interacts Directly with AT_1_R, AT_2_R, and MasR

Naïve HEK-293T cells do not express functionally significant concentrations of RAS components. As such, we hypothesize that outcomes associated with ACE2 expression may be a direct result of receptor-enzyme interactions.

**Figure 4:**
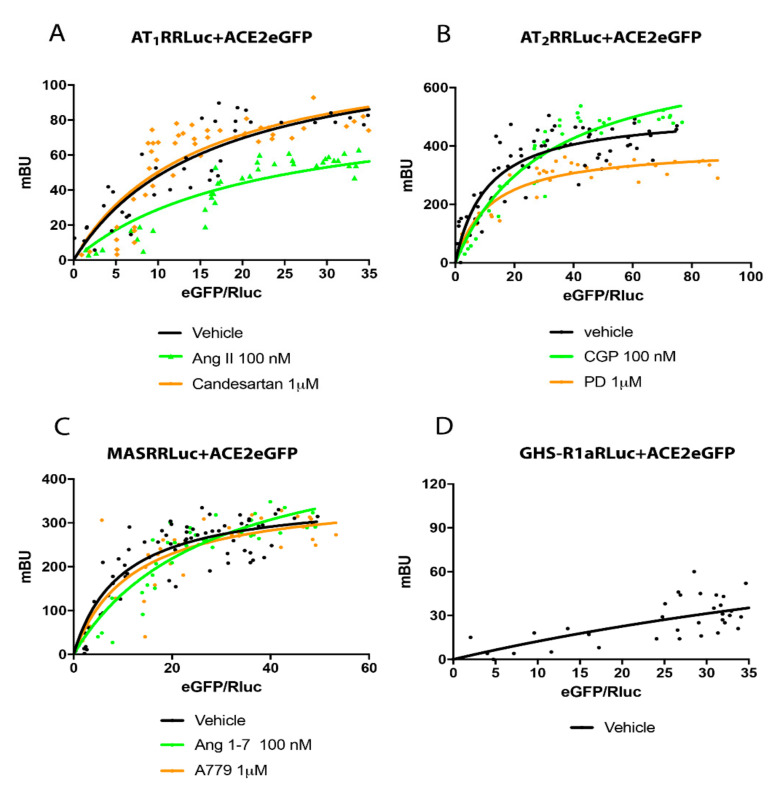
Interactions of RAS receptors and ACE2 as assessed by Bioluminescence Resonance Energy Transfer (BRET) assays. BRET assays were performed in HEK-293T cells transfected with constant amounts of cDNA encoding AT_1_R-RLuc (0.5 µg) (**A**), AT_2_R-RLuc (0.4 µg) (**B**), MasR-RLuc (0.6 µg) (**C**), or GHS-R1a-RLuc (0.3 µg; negative control) (**D**) together with increasing amounts of cDNA encoding ACE2-eGFP (0.1 to 1 µg). Cells were treated (red symbols) or not (black symbols) for 25 min with selective antagonists (candesartan for AT_1_R, PD123319 for AT_2_R or A779 for MasR, both at 1 µM; red symbols) or selective agonists (Ang II for AT_1_R, CGP for AT_2_R or Ang 1-7 for MasR, all at 100 nM; green symbols). Values correspond to experimental points from 6 independent experiments each performed in quadruplicate. BRET_50_ and BRET_max_ values were calculated by non-linear regression using Prism GraphPad software; specific parameters are as described in the text.

This hypothesis was tested using BRET assays. First, HEK-293T cells were transfected with a constant amount of cDNA encoding AT_1_R-RLuc and increasing amounts of cDNA encoding ACE2-eGFP. Findings shown in Figure 4A include a saturable BRET curve which is indicative of direct interactions between AT_1_R and ACE2 (BRET_max_ = 120 ± 20 mBU and BRET_50_ = 16 ± 4). Similar assays were performed in cells pretreated for 10 min with Ang II or with the selective AT_1_R antagonist, candesartan. While a significant decrease in BRET signal was detected after challenging with the agonist (BRET_max_ = 90 ± 10 mBU and BRET_50_ = 21 ± 5), these parameters did not undergo significant change in response to treatment with a receptor antagonist, candesartan (BRET_max_ = 12 ± 20 mBU and BRET_50_ = 15 ± 5; Figure 4A). These results may be explained by conformational changes that alter the distance between BRET donor and BRET acceptor. Another possibility is internalization, disassembly and recycling in response to agonist challenge. The latter process would lead to reduce the number of AT_1_R-ACE2 complexes.

Potential AT_2_R-ACE2 interactions were examined in experiments performed in HEK-293T cells transfected with a constant amount of cDNA encoding AT_2_R-RLuc and increasing amounts of cDNA encoding ACE2-eGFP. The saturable BRET curve (BRET_max_ = 510 ± 20 mBU and BRET_50_ = 11 ± 2) revealed the formation of AT_2_R-ACE2 complexes in the co-transfected HEK-293T cells. However, we observed a significant increase in the height at saturation (BRET_max_ = 750 ± 70 mBU and BRET_50_ = 31 ± 6) in co-transfected cells that were challenged with the AT_2_R agonist, CGP (Figure 4B). These results may be explained by conformational changes that reduce the distance between the BRET donor and BRET acceptor. The findings might also be explained by an increase in the number of receptor-enzyme complexes. Challenge with the AT_2_R antagonist, PD123319, led to a significant decrease in the BRET signal when compared to results from untreated cells (BRET_max_ = 400 ± 20 mBU and BRET_50_ = 12 ± 2).

Finally, we addressed the possibility of physical interactions between MasR and ACE2. BRET assays confirmed these interactions. However, we observed no responses to treatment with MasR agonists or antagonists (Figure 4C). Parameters defining this interaction were: BRET_max_ = 362 ± 21 mBU and BRET_50_ = 10 ± 2 in the absence of receptor activation, BRET_max_ = 420 ± 60 mBU and BRET_50_ = 16 ± 5 in cells treated with receptor agonist, and BRET_max_ = 370 ± 40 mBU and BRET_50_ = 12 ± 4 in cells treated with receptor antagonist. A non-specific linear signal was obtained in HEK-293T cells that were transfected with the cDNA encoding GHS-R1a-RLuc (negative control) together with increasing amounts of ACE2-eGFP (Figure 4D).

### 2.5. Cell Surface Expression of ACE2 Following Activation of AT_1_R and AT_2_R

Our findings revealed that ACE2 was capable of functionally interact with RAS receptors. We also discovered that direct interactions between ACE2 and AT_1_R and AT_2_R underwent a quantitative change in response to receptor activation. As such, our next aim was to assess cell surface expression of ACE2 following RAS receptor activation.

**Figure 5:**
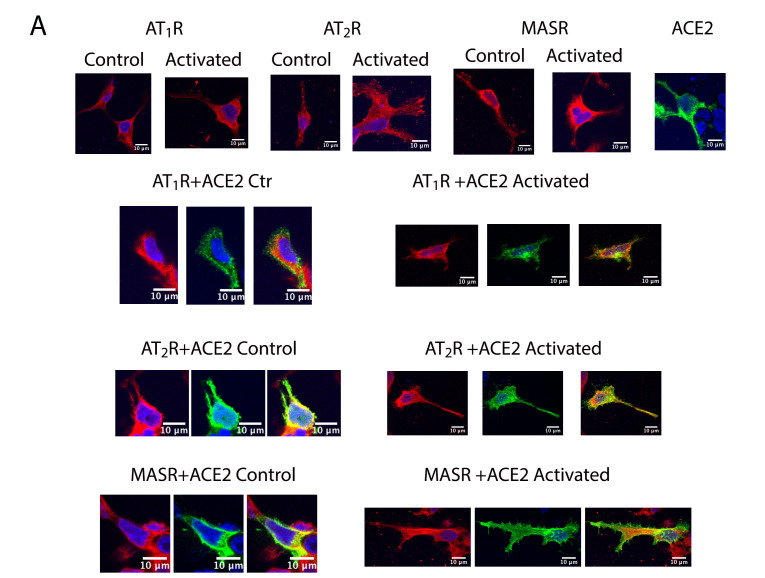

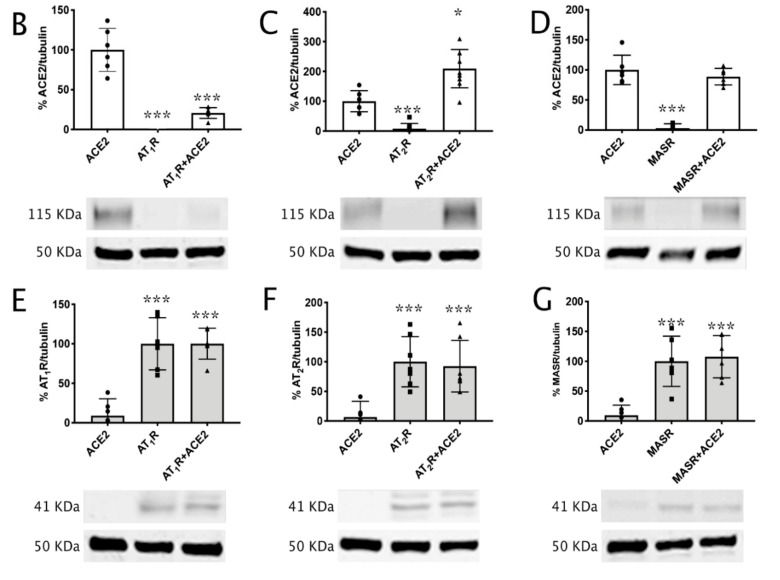
RAS receptors regulate cell surface expression of ACE2. (**A**) Immunocytochemistry assays were performed in HEK-293T cells expressing ACE2-eGFP together with AT_1_R-RLuc, AT_2_R-RLuc, or MasR-RLuc and activated with the respective agonists. Cells that expressed each RAS receptor were treated with their respective selective agonist. ACE2-eGFP expression was evaluated quantitatively via green fluorescence. The RLuc-containing receptors were detected by an anti-RLuc primary antibody and a secondary Cy3-conjugated anti-mouse antibody (red-staining). Colocalization is shown in yellow. Scale bar = 10 µm. (**B**–**G**) Biotinylation experiments were performed in HEK-293T cells transfected with cDNA encoding AT_1_R-RLuc (1 µg; **B**,**E**), AT_2_R-RLuc (1 µg; **C**,**F**) or MasR-RLuc (1 µg; **D**,**G**) with or without 0.8 μg of ACE2-HA cDNA. Images from a representative experiment are shown (expansion of the image areas in Figure 5 and position of the MW from this representative experiment appear in Supplementary Figure S1). Immunoreactive bands from 6 independent experiments were quantified. Values presented are the mean ± SD. One-way ANOVA followed by Bonferroni’s multiple comparison posthoc tests were used for statistical analysis; * *p* < 0.05, *** *p* < 0.001 vs. ACE2-HA singly-transfected cells.

Cell surface expression of ACE2 was first assessed by immunocytochemistry assays targeting HEK-293T cells that express this enzyme together with AT_1_R, AT_2_R, or MasR. As shown in Figure 5A, ACE2 and the three receptors were all detected at the plasma membrane and were also associated with intracellular structures in the cytoplasm. Dual localization was also observed in cells treated with selective agonists.

Increased green fluorescence documenting ACE2 immunoreactivity in the cytoplasm was detected in AT_1_R and ACE2-expressing cells that were treated with Ang II. These results suggested that activation of AT_1_R results in decreased expression of ACE2 at the plasma membrane (Figure 5A). By contrast, no significant changes in ACE2 immunoreactivity were detected when cells expressing AT_2_R or MasR were treated with their respective receptor agonists.

For improved assessment of cell surface expression, we performed biotinylation experiments using co-transfected cells. The assay conditions used in these experiments permit biotinylation of cell surface proteins only. Immunoblotting was performed on preparations of isolated biotinylated proteins. Fusion proteins containing Rluc or eYFP were used to normalize protein expression; this facilitated comparisons of similar expression levels in experiments performed in cells that expressed ACE2 alone, a receptor, or ACE2 together with a receptor. Using this method, we found that co-expression of ACE2 had no significant impact on the expression of any of the three RAS receptors evaluated (Figure 5B–G).

The results from HEK-293T cells that co-expressed AT_1_R-RLuc and ACE2 and were activated with Ang II revealed decreased expression of ACE2 on Western blots (Figure 5B). When similar assays were performed in cells that co-expressed AT_2_R-RLuc and ACE2, a significant increase in the enzyme cell surface expression was observed (Figure 5C). However, no differential regulation of ACE2 was observed in HEK-293T cells co-expressing MasR (Figure 5D). The presence of ACE2 had no impact on the expression of AT_1_R, AT_2_R, or MasR (Figure 5E–G).

Taken together, our results indicate that ligand-mediated activation of AT_1_R resulted in diminished cell surface expression of ACE2. By contrast, activation of AT_2_R resulted in elevated levels of ACE2. No differential expression of this enzyme was observed in response to activation of MasR.

### 2.6. Detection of AT_1_R-ACE2, AT_2_R-ACE2, and MasR-ACE2 Complexes in the Lungs of Adult Mice

Children are less likely to succumb to severe SARS-CoV2 infection; it has been hypothesized that this may be due to comparatively lower levels of ACE2 in lung tissue. Given the findings described above, our next goal was to evaluate ACE2-receptor complexes in the lungs of adult and fetal CD-1 mice. This investigation was approached using sections of fixed tissue and in situ proximity ligation assay (PLA). This technique is instrumental for the detection of complexes formed by two proteins in natural sources.

**Figure 6:**
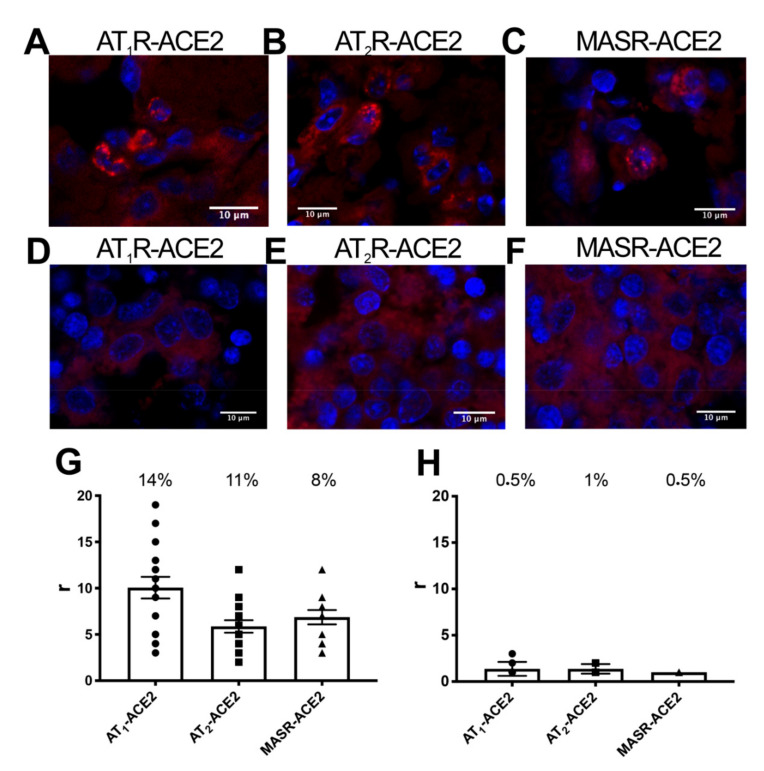
Detection of AT_1_R-ACE2, AT_2_R-ACE2, and MasR-ACE2 complexes in mouse lung tissue. Proximity Ligation Assays (PLAs) were performed using lung sections of adult (**A**–**C**) or 19-day fetal CD-1 mice (**D**–**F**) as described in the Methods. Cell nuclei were stained with Hoechst (blue). Protein complexes appear as red clusters or dots. Representative images corresponding to stacks of 4 sequential planes are shown. Graphs (**G**,**H**) display the number of clusters and spots in spot-containing cells. Values presented are the mean ± SEM of 5 independent experiments.

Using specific antibodies raised against the proteins of interest, we could detect that 14% of cells expressed AT_1_R-ACE2 complexes with around 10 red dots/cell-expressing dots (Figure 6A), 11% of cells expressed AT_2_R-ACE2 complexes with around 6 red dots/cell-expressing dots (Figure 6B) and that only 8% of cells expressed MasR-ACE2 complexes with around 7 red dots/cell-expressing dots (Figure 6C). These results demonstrate the existence of AT_1_R-ACE2, AT_2_R-ACE2 and MasR-ACE2 complexes in the adult lung of mice (Figure 6G). Remarkably, no positive signals were observed for any pair of proteins in sections from fetuses (<1% of cells displayed red clusters) (Figure 6D–F,H).

## 3. Discussion

ACE2 on target cells has been identified as a critical receptor for SARS and other coronaviruses [23,24,25,26]. However, the mechanisms underlying SARS-CoV-2 entry into cells and the role of ACE2 in facilitating viral infection remain to be clarified. Similar to what has been observed for HIV-1 infection, GPCRs may facilitate viral infection. GPCRs constitute approximately 10% of the human proteome; they are typically expressed on the cell surface and can be internalized via clathrin- or caveolin-dependent mechanisms [27,28,29,30]. To the best of our knowledge, there are no published studies that link GCPRs or GPCR internalization mechanisms to the coronavirus life cycle. Of note, peptides derived from SARS, HIV-1 and Ebola virus proteins bind with high affinity to formyl-peptide receptors [30,31]. The physiological role of this GPCR with respect to viral infection is currently unknown.

The chemokine receptor CXCR4 was identified as an HIV-1 co-receptor and mediator of cell entry soon after the identification of this pathogen as the causative agent of AIDS [13,16]. Subsequent studies revealed that CD26/DPPIV interacts with CXCR4 and is targeted by envelope gp120 HIV-1 glycoprotein [19,22,32,33]. HIV-1 entry into target cells involves CXCR4 as well as interactions with CD26/DPPIV and the receptor agonist, CXCL12/SDF-1, which is also a substrate of this enzyme. Potential parallels to mechanisms underlying HIV-1 infection have led us to hypothesize that there is a gap in SARS-related research that will be only fulfilled once GPCRs are considered [34].

As the most straightforward approach toward the identification of GPCRs that may be involved in SARS-CoV-2 infection, we focused on receptors that interact with the ACE2 substrate, Ang II. Toward this end, results of RNA-based interference assays performed in the dorsal vagal complex of the mouse brainstem suggested the existence of interactions between ACE2 and AT_1_R [35]. However, the approach used in this study was not designed to address the possibility of direct protein-protein interactions. Deshotels et al. [36] performed an important study using both cell transfection and rodent model approaches that revealed Ang II-mediated and AT_1_R-dependent regulation of ACE2 expression; the results of co-immunoprecipitation experiments suggested interactions between the two cell surface proteins. This biophysical approach here used, BRET, allows identification of direct interactions; the results revealed that ACE2 may interact with both AT_1_R and AT_2_R and also with MasR, which is the receptor for Ang 1-7, the product of ACE2-mediated cleavage of Ang II.

Protein-protein interactions occurring at the plasma membrane can modify receptor-mediated signaling responses. As shown here, the expression of ACE2 modulated agonist-induced signaling at all three receptors (i.e., AT_1_R, AT_2_R, or MasR). Negative modulation of AT_1_R or AT_2_R-mediated signaling might be expected, given that ACE2 ultimately degrades the endogenous agonist, Ang II. However, the high-affinity CGP agonist used to promote signaling via AT_2_R does not undergo significant degradation by ACE2. Equally interesting was the modulation of ACE2 expression observed upon receptor activation. Our results add to findings reported by Bai et al. [37], who described telmisartan-induced downregulation of AT_1_R with a concomitant increase in ACE2 activity; treatment of hypertensive rats with this selective AT_1_R antagonist rebalanced the RAS system. We found that activation of MasR, the receptor for Ang 1-7, had no significant impact on ACE2 expression. However, activation of the Ang II receptors, AT_1_R and AT_2_R, led to different outcomes, a finding that is consistent with the opposing physiological roles presumably played by these two receptors.

The loss of cell surface ACE2 in a physiological context could be due to endocytosis or to shedding; the latter phenomenon has been reported for the HIV-1-related peptidase, CD26/DPPIV [20,21]. However, in our experimental conditions, changes in cell surface expression of ACE2 were largely dependent on processes underlying endocytosis and trafficking to the cell surface. This finding is important as it implies that the availability of the SARS receptor might vary depending on the status of the RAS, most notably on the local concentration of Ang II and the relative expression of both AT_1_R and AT_2_R. As these parameters may vary from cell to cell even under homeostatic conditions, SARS-CoV-2 infection may generate symptoms of varying severity. In other words, the expression of RAS components in a given cell may dictate its susceptibility to SARS-CoV-2 infection and responses that include mild to more severe symptoms of this disease. Our results suggest that cells that express higher levels of AT_2_R, especially when activated by Ang II, are more likely to facilitate SARS-CoV-2 attachment due to higher levels of ACE2 expression. Further experiments, preferably those performed with infectious SARS-CoV-2 particles, would be needed to determine whether cells enriched in AT_2_R and activated by the endogenous agonist are more susceptible to virion binding, and to assess whether the RAS-related GPCRs complexed with ACE2 promote viral endocytosis.

Severe COVID-19 involves pneumonia and fatal outcomes that often correlate with elevated plasma levels of interleukin-6 (IL-6) and the cytokine storm [38,39,40,41,42]. High levels of IL-6 may be due to its overproduction by activated macrophages as well as by non-immune cells. There has been reported that bacterial endotoxins induce the synthesis of IL-6 in osteoblasts [43] and endothelial cells [44]. High levels of ACE2 expression have been detected in the lung which includes an air-exposed interface that is composed of a variety of cells; ACE2 in the human lung is particularly abundant in bronchial transient secretory cells [45,46]. Lung tissue includes both endothelial and epithelial cells that can express nearly all RAS components. Of particular note, ACE2 has been localized at the apical side of polarized cells [47] where it can facilitate interactions with inhaled virions. As such, our final important objective was to identify ACE2-receptor complexes in the lung. This was made possible by in situ PLA, which is a technique that was specifically developed to detect complexes formed by two membrane proteins [48]. Using this method, we found that ~10% of the cells in adult mouse lung tissue expressed one or more complexed protein pairs, including ACE2/AT_1_R, ACE2/AT_2_R, and ACE2/MasR. Remarkably, no complexes were detected in mouse fetal lung sections. Given these findings, it is tempting to speculate that the predominance of mild or even asymptomatic infection in children exposed to SARS-CoV-2 might be directly related to the absence of receptor-enzyme complexes that promote viral entry into lung cells. Taken together, our findings suggest that further consideration of ACE2-containing RAS receptor complexes might reveal critical features underlying the mechanism of SARS-CoV-2 infection.

## 4. Material and Methods

Studies were designed to include groups of equal size and used randomization methods and blinded analysis. Antibody-based immunocytochemical assays were conducted in line with guidelines detailed elsewhere [49,50].

### 4.1. Reagents

Ang II, CGP, Ang 1-7, candesartan, PD123319, A779, and forskolin were purchased from Sigma-Aldrich (St. Louis, MO, USA).

### 4.2. Expression Vectors

cDNAs encoding human AT_1_R, AT_2_R, and MasR were amplified without their stop codons using sense and antisense primers that included either BamHI and HindIII restriction sites (for amplification of AT_1_R and AT_2_R) or BamHI and EcoRI restriction sites (for amplification of MasR) and subcloned into the pcDNA3.1 expression vector. Amplified fragments were subcloned in-frame with genes encoding the enhanced yellow fluorescent protein (pEYFP-N1; Clontech, Heidelberg, Germany) or RLuc (pRluc-N1; PerkinElmer, Wellesley, MA, USA) at the respective C-termini to produce AT_1_R-RLuc, AT_1_R-YFP, AT_2_R-RLuc, AT_2_R-YFP, MAS-RLuc, and MAS-YFP fusion proteins. cDNAs in pcDNA3.1 for ACE2-eGFP (ACE2_OHu20260C_pcDNA3.1(+)-C-eGFP Clone ID: OHu20260C; ORF Clones: Accession No.:NM_021804.3) and for ACE2-HA (ACE2_OHu20260C_pcDNA3.1(+)-N-HA Clone ID:OHu20260C; ORF Clones: Accession No.:NM_021804.3) were purchased from GenScript Biotech (Leiden, The Netherlands).

### 4.3. Cell Culture and Transient Transfection

HEK-293T cells were grown in Dulbecco’s Modified Eagle’s Medium (DMEM; Gibco, Paisley, Scotland, UK) supplemented with 2 mM L-glutamine, 100 U/mL penicillin/streptomycin, MEM Non-Essential Amino Acids Solution (1/100) and 5% (*v/v*) heat-inactivated fetal bovine serum (FBS) (Invitrogen, Paisley, Scotland, UK). Cells were maintained in a humid atmosphere at 5% CO_2_ at 37 °C. Cells were transiently transfected using the polyethylenimine (PEI; Sigma-Aldrich) method as previously described [51].

### 4.4. Bioluminescence Resonance Energy Transfer (BRET) Assays

HEK-293T cells were transiently transfected with a constant amount of cDNA encoding AT_1_R-RLuc, AT_2_R-RLuc, MasR-RLuc, or GHS-R1a-RLuc (the latter used as a negative control) together with increasing amounts of cDNA encoding ACE2-eGFP. At 48 h after transfection, cells were adjusted to 20 μg of protein using a Bradford assay kit (Bio-Rad, Munich, Germany) using bovine serum albumin (BSA) for standardization. To quantify protein-eGFP expression, fluorescence was read in a FluoStar Optima Fluorometer (BMG LabTechnologies, Offenburg, Germany) equipped with a high-energy xenon flash lamp and a 10 nm bandwidth excitation filter at 485 nm. For BRET measurements, readings were collected 30 s after the addition of 5 μM coelenterazine H (Molecular Probes, Eugene, OR, USA) using a Mithras LB 940, which facilitates the integration of signals detected from both the short-wavelength (485 nm) and the long-wavelength filters (510 nm). To quantify protein-RLuc expression, luminescence readings were performed 10 min after the addition of 5 μM coelenterazine H also using a Mithras LB 940. The net BRET is defined as ([long-wavelength emission]/[short-wavelength emission]) minus C_f_, with C_f_ corresponding to the [long-wavelength emission]/[short-wavelength emission] ratio for the donor construct expressed alone in the same experiment. GraphPad Prism software (San Diego, CA, USA) was used to fit the data. BRET is expressed as milli-BRET units (mBU = net BRET × 1000).

### 4.5. Immunostaining Procedures

HEK-293T cells expressing AT_1_R-RLuc, AT_2_R-RLuc, or MasR-RLuc in the presence or the absence of ACE2-eGFP were fixed in 4% paraformaldehyde for 15 min and washed twice with phosphate-buffered saline (PBS) containing 20 mM glycine (PBS-glycine). Washed cells were then permeabilized with PBS-glycine containing 0.5% Triton X-100 for 5 min. Fixed and permeabilized cells were treated for 1 h with PBS containing 1% BSA and labeled with primary mouse anti-RLuc antibody (1/100; Millipore, MA, USA) followed by secondary Cy3-conjugated anti-mouse IgG antibody (1/200; Jackson ImmunoResearch, Philadelphia, PA, USA). Samples were washed several times and mounted with 30% Mowiol (Calbiochem/Merck Group, Darmstadt, Germany). Samples were observed using a Zeiss 880 confocal microscope. The signal from eGFP was detected by its green fluorescence and could be distinguished from the red signal observed in response to binding of Cy3-conjugated anti-mouse IgG. Colocalization was identified by yellow fluorescence. The scale bar presented in images measured 10 µm.

### 4.6. cAMP Determination

HEK-293T cells were transfected with cDNAs encoding AT_1_R, AT_2_R, or MasR in the presence or the absence of ACE2-HA. Cells were serum-starved in DMEM alone for 2 h before initiating an experiment. Starved cells were detached and suspended in culture medium containing 50 µM of the phosphodiesterase inhibitor, zardaverine (Sigma-Aldrich). Cells were then distributed into 384-well microplates at 2500 cells/well and stimulated for 15 min with increasing concentrations of agonists, including Ang II for AT_1_R, CGP for AT_2_R, and Ang 1-7 for MasR. Agonists were added at concentrations 0.1 nM to 3 µM or vehicle alone; this was followed by the addition of 0.5 µM forskolin or vehicle alone for an additional 15 min. Readings were performed after a 1 h incubation at 25 °C. Homogeneous time-resolved fluorescence energy transfer (HTRF) measurements were performed using the Lance Ultra cAMP kit (PerkinElmer, Waltham, MA, USA). Fluorescence at 665 nm was analyzed on a PHERAstar Flagship microplate reader equipped with an HTRF optical module (BMG Lab Technologies, Offenburg, Germany).

### 4.7. ERK Phosphorylation Assays

To determine the extent of ERK1/2 phosphorylation, HEK-293T cells were transfected with cDNAs encoding AT_1_R, AT_2_R, or MasR in the presence or the absence of ACE2-HA, plated at 40,000 cells/well in transparent Deltalab 96-well microplates, and incubated at 5% CO_2_ for 48 h. Cells were serum-starved in DMEM alone for 2–4 h before the start of the experiment. Serum-starved cells were treated for 7 min at 25 °C with vehicle or increasing concentrations of agonists (0.1 nM to 3 µM) including Ang II, CGP, or Ang 1-7 for specific receptors as described above. Cells were then washed twice with cold PBS and placed in lysis buffer; cells were lysed for 20 min while undergoing agitation. A 10 μL aliquot of each supernatant was placed in each well of a white ProxiPlate 384-well microplate, and the degree of ERK 1/2 phosphorylation was determined using an AlphaScreen^®^SureFire^®^ kit (Perkin Elmer) following the instructions of the supplier and using an EnSpire^®^ Multimode Plate Reader (PerkinElmer).

### 4.8. β-Arrestin 2 Recruitment

Recruitment of β-arrestin was evaluated as previously described [52,53]. Briefly, Bioluminescence Resonance Energy Transfer (BRET) experiments were performed using HEK-293T cells at 48 h after transfection with cDNAs encoding AT_1_R-YFP, AT_2_R-YFP, or MasR-YFP together with β-arrestin II-RLuc in the presence or the absence of ACE2-HA. Cells (20 μg protein per aliquot) were distributed in 96-well microplates (Corning 3600, white plates with white bottom, Sigma-Aldrich) and were stimulated for 10 min with the indicated agonists, including Ang II, CGP, or Ang 1-7 as described above at concentrations of 0.1 nM to 3 µM before the addition of 5 μM coelenterazine H (Molecular Probes, Eugene, OR). Within 1 min of the addition of coelenterazine H, BRET between β-arrestin II-RLuc and receptor-YFP was determined and quantified. The readings were collected using a Mithras LB 940 (Berthold Technologies, Bad Wildbad, Germany) that facilitates the integration of the signals as described above. To quantify protein-RLuc expression, luminescence readings were performed at 10 min after the addition of 5 μM coelenterazine H.

### 4.9. Cytoplasmic Ca^2+^ Detection

HEK-293T cells were transfected with the cDNA encoding AT_1_R, AT_2_R, or MasR together with the Ca^2+^ sensor, GCaMP6 [54] in the presence or the absence of ACE2-HA using the PEI method. At 24 h after transfection, 150,000 cells were plated in each well of a 96-well black, clear-bottom microtiter plate. Cells were then incubated with Mg^+2^-free Locke’s buffer (154 mM NaCl, 5.6 mM KCl, 3.6 mM NaHCO_3_, 2.3 mM CaCl_2_, 5.6 mM glucose, and 5 mM HEPES at pH 7.4) supplemented with 10 μM glycine, and pre-treated for 10 min with the selective antagonists, including candesartan (for AT_1_R), PD123319 (for AT_2_R) and A779 (for MasR), followed by stimulation with increasing concentrations (1 nM to 1 µM) of agonists, including Ang II, CGP, and Ang 1-7 as described above. The fluorescence emission intensity of GCaMP6 was recorded (every 5 s for 150 s, 100 flashes/well) at 515 nm upon excitation at 488 nm on the EnSpire^®^ multimode plate reader.

### 4.10. Dynamic Mass Redistribution (DMR) Assays

Cell mass redistribution induced upon receptor activation was detected by illuminating the underside of the biosensor with polychromatic light followed by measuring the changes in the wavelength of the reflected monochromatic light. HEK-293T cells expressing AT_1_R, AT_2_R, or MasR in the presence or the absence of ACE2-HA were seeded in 384-well sensor microplates to 70–80% confluency (approximately 10,000 cells per well). Before the start of the experiment, cells were washed twice with assay buffer (Hank’s buffered saline solution [HBSS] with 20 mM HEPES, pH 7.15) followed by a 2 h incubation with assay-buffer containing 0.1% dimethyl sulfoxide (DMSO; 24 °C, 30 μL/well). The sensor plate was then scanned, and a baseline optical signature was recorded for 10 min before adding 10 μL of each of the specific antagonists (candesartan, PD123319, or A779 as described above). Responses were recorded for 30 min; this was followed by the addition of 10 μL of increasing concentrations of the selective agonists (Ang II, CGP, or Ang 1-7) at concentrations from 1 nM to 1 μM; all test compounds were dissolved in assay buffer. Dynamic mass distribution (DMR) responses were monitored for at least 3000 s using an EnSpire^®^ Multimode Plate Reader (PerkinElmer). Results were analyzed using EnSpire Workstation Software v 4.10.

### 4.11. Immunoblotting

To determine levels of immunoreactive AT_1_R, AT_2_R, MasR, and ACE2-HA expression in transfected HEK-293T cells, equivalent amounts of cell protein (10 μg) were separated by denaturing 10% sodium dodecyl sulfate (SDS)-polyacrylamide gel electrophoresis (PAGE) and transferred onto polyvinylidene difluoride (PVDF)-fluorescence membranes. Membranes were treated overnight at 4 °C with a mixture of a mouse anti-β-tubulin antibody (1:2000; Sigma-Aldrich), a rabbit anti-ACE2 antibody (1:1000; Cat# ab108252, RRID:AB_10864415, Abcam, Cambridge, UK) and a mouse monoclonal anti-AT_1_R antibody (1:1000; Cat# sc-515884, RRID:AB_2801404, Santa Cruz Biotechnology, Dallas, TX, USA), a rabbit monoclonal anti-AT_2_R antibody (1:1000; Cat# ab92445, RRID:AB_10561969, Abcam, Cambridge, UK) and a mouse monoclonal anti-MasR antibody (1:1000; Cat# sc-390453, RRID:AB_2801406, Santa Cruz Biotechnology). Cells were subsequently treated with a mixture of IRDye 800-conjugated anti-mouse antibody (1:10,000; #A9044 from Sigma-Aldrich) and IRDye 680-conjugated anti-rabbit antibody (1:10,000; #926-68071 from LICOR Biosciences, Lincoln, NE, USA) for 2 h at room temperature. Bands were scanned using the Odyssey infrared scanner (LI-COR Biotechnology, Lincoln, NE, USA). Band densities were quantified using the scanner software and receptor level was normalized for differences in loading via normalization to tubulin protein band intensities.

### 4.12. Biotinylation Experiments

Cell surface proteins were biotinylated as previously described [55,56] using HEK-293T cells that transiently express AT_1_R-RLuc, AT_2_R-RLuc, or MasR-RLuc in the presence or the absence of ACE2-HA. Before initiation of the experiment, eGFP fluorescence was adjusted to 10,000 fluorescence units and receptor-RLuc to 100,000 bioluminescent units. Briefly, cells were washed three times with borate buffer (10 mM H_3_BO_3_, pH 8.8 with 150 mM NaCl) and incubated with 50 µg/mL sulfo-NHS-LC-biotin (ThermoFisher Scientific, Halethorpe, MD, USA) in borate buffer for 5 min at room temperature. Cells were then washed three times in borate buffer and again incubated with 50 µg/mL sulfo-NHS-LC-biotin in borate buffer for 10 min at room temperature. This was followed by the addition of 13 mM NH_4_Cl for 5 min to quench the remaining biotin. Cells were washed in PBS, disrupted using a polytron (3 strokes at 10 s each), and centrifuged at 16,000× *g* for 30 min. The pellet was solubilized in an ice-cold RIPA buffer (50 mM Tris–HCl, 1% Triton X-100, 0.2% SDS, 100 mM NaCl, 1 mM EDTA, 0.5% sodium deoxycholate) for 30 min and centrifuged at 16,000× *g* for 20 min. The supernatant was incubated with 80 µl streptavidin-agarose beads (Sigma-Aldrich) for 1 h with constant rotation at 4 °C. Beads were washed three times with ice-cold lysis buffer and aspirated to dryness using a 28-gauge needle. Subsequently, 50 µl of SDS–PAGE sample buffer (8 M urea, 2% SDS, 100 mM dithiothreitol, 375 mM Tris, pH 6.8) was added to each sample. Proteins were dissociated by heating to 37 °C for 2 h, resolved by SDS-PAGE (10% gels), and immunoblotted as described above.

### 4.13. In Situ Proximity Ligation Assays (PLA)

Lungs from adult and 19-day-old fetal CD-1 mice were fixed in 4% paraformaldehyde for 1 day followed by processing with decreasing concentrations of sucrose. Tissue samples were cut in 30 µm-thick sections in a cryostat (Leica CM3050S), mounted on coverslips, and frozen. Frozen tissue samples were washed with PBS containing 20 mM glycine and permeabilized by incubation for 30 min in PBS-glycine containing 0.05% Triton X-100. Tissue sections were incubated for 1 h at 37 °C with blocking solution followed by specific antibodies, including mouse anti-AT_1_R (1:100; Cat# sc-515884, RRID:AB_2801404, Santa Cruz Biotechnology), rabbit anti-AT_2_R (1:100; Cat# ab92445, RRID:AB_10561969, Abcam), mouse anti-MasR (1:100; Cat# sc-390453, RRID:AB_2801406, Santa Cruz Biotechnology), and rabbit anti-ACE2 (1:100; Cat# ab108252, RRID:AB_10864415, Abcam). These samples were processed using PLA probes that detect rabbit and mouse antibodies (Duolink II PLA probe anti-Rabbit plus and Duolink II PLA probe anti-Mouse minus). Nuclei were stained with Hoechst (1/200; Sigma-Aldrich) and mounted in 30% Mowiol (Calbiochem). Samples were observed using a Zeiss 880 confocal microscope (Leica Microsystems, Mannheim, Germany) equipped with an apochromatic 63× oil-immersion objective (N.A. 1.4), and 405 nm and 561 nm laser lines. For each field of view, a stack of two channels (one per staining) and three to four Z stacks with a step size of 1 µm were acquired. Duolink Image took software was used to identify cells with one or more red spots vs. total cells (cell count determined by the presence of a single blue-stained nucleus). The ratio r was determined as the number of red spots per cell in all red spot-containing cells. This analysis was performed in a blinded fashion (i.e., the observer did not know which sample was undergoing processing and the analyzer did not know whether the results came from adult or fetal mice.

### 4.14. Validation of Antibody Specificity

Despite the excellent performance of these antibodies in different laboratories [57,58,59,60], the specificity of antibodies directed against angiotensin receptors is always subject to question. As such, we performed a series of experiments in which the anti-Ang II receptor antibodies were tested against naïve HEK-293T cells and against cells that express either AT_1_R or AT_2_R. Signal detected from anti-AT_1_R antibody binding was negligible in both naïve and AT_2_R-expressing HEK-293T cells. Similarly, the signal detected from anti-AT_2_R antibody binding was negligible in both naïve and AT_1_R-expressing cells (Supplementary Figure S2). These results are consistent with previous studies that addressed the specificity of antibodies used to detect AT_1_Rs in mitochondria [61].

### 4.15. Data Analysis

Data were obtained from at least five independent experiments and are presented as the mean ± standard error of the mean (SEM). Two-group comparisons were performed using unpaired Student’s *t*-tests. Multiple comparisons were performed using one-way ANOVA followed by Bonferroni’s post hoc test or two-way ANOVA followed by Tukey’s post hoc test. The normality of populations and homogeneity of variances were tested before performing ANOVA. Post hoc tests were run only in the cases in which F achieved *p* < 0.05 and in which there was no significant variance with respect to homogeneity. Statistical analysis was undertaken only when each group size was at least *n* = 5, with n representing the number of independent variables. Technical replicates were not treated as independent variables. Unequal group sizes were due to (a) different sources due to the wide variety of experimental approaches, (b) the need to increase the “n” to ensure data reliability in some of the assays, (c) animal availability, and/or (d) economy of resources as directed by the 3Rs (Replacement, Reduction, and Refinement) rule that governs experimentation with animals. Differences were considered significant when *p* ≤ 0.05. Statistical analyses were carried out with GraphPad Prism software version 5 (San Diego, CA, USA; (RRID: SCR_002798)). Outlier tests were not used; all data points (representing the means of technical replicates) were used for analysis. The data and statistical analysis comply with the recommendations detailed elsewhere [49].

### 4.16. Nomenclature of Targets and Ligands

Key protein targets and ligands in this article are hyperlinked to corresponding entries in http://www.guidetopharmacology.org, the common portal for data from the IUPHAR/BPS Guide to PHARMACOLOGY [62] and are permanently archived in the Concise Guide to PHARMACOLOGY 2019/20 [6].

## Supplementary Materials

The following are available online at https://www.mdpi.com/1422-0067/21/24/9602/s1, Figure S1: Expansion of the image areas in Figure 5 and position of the MW markers corresponding to immunoblots in Figure 5 of the main paper (panels refer to those in Figure 5). Figure S2: Antibody specificity control assays.

## Declaration of Transparency and Scientific Rigor

This Declaration acknowledges that this paper adheres to the principles for transparent reporting and scientific rigor for preclinical research as recommended by our Institutions, funding agencies, publishers, and other organizations engaged in supporting research.
