# Supplementary material for: Functional Complexes of Angiotensin-Converting Enzyme 2 and Renin-Angiotensin System Receptors: Expression in Adult but Not Fetal Lung Tissue
Source: Int J Mol Sci. 2020 Dec 16;21(24):9602. doi: 10.3390/ijms21249602 (PMC7766085; doi:10.3390/ijms21249602)
Supplement: Supplementary file 1 [file ijms-21-09602-s001.pdf]

International Journal of *Molecular Sciences*

MDPI

Supplementary material

Discovery of functional complexes of angiotensin converting enzyme 2 and renin angiotensin system receptors. Expression in the adult, but not in the fetal lung

Rafael Franco[a,b], Alejandro Lillo[a], Rafael Rivas-Santisteban[a,b], Ana I. Rodríguez-Pérez[b,c], Irene Reyes-Resina[a,d], José L. Labandeira-García[b,c], Gemma Navarro[b,e]

[a] Laboratory of Molecular Neurobiology. Department Biochemistry and Molecular Biomedicine. School of Biology. University of Barcelona. Barcelona (Catalonia). Spain.

[b] Centro de Investigación en Red, Enfermedades Neurodegenerativas (CIberNed). Instituto de Salud Carlos iii. Madrid (Madrid). Spain.

[c] Laboratory of Cellular and Molecular Neurobiology of Parkinson's disease, Research Center for Molecular Medicine and Chronic Diseases (CIMUS), Dept. of Morphological Sciences, IDIS, University of Santiago de Compostela, Santiago de Compostela; Spain.

[d] Present address. RG Neuroplasticity, Leibniz Institute for Neurobiology, 39118 Magdeburg, Germany.

[e] Department of Biochemistry and Physiology. School of Pharmacy and Food Science. University of Barcelona. Barcelona (Catalonia). Spain.

Correspondence: rfranco@ub.edu ; Tel.: +34-934-021-208 (R.F.); dimartts@hotmail.com; Tel.: + 34-934-034-500 (G.N.)

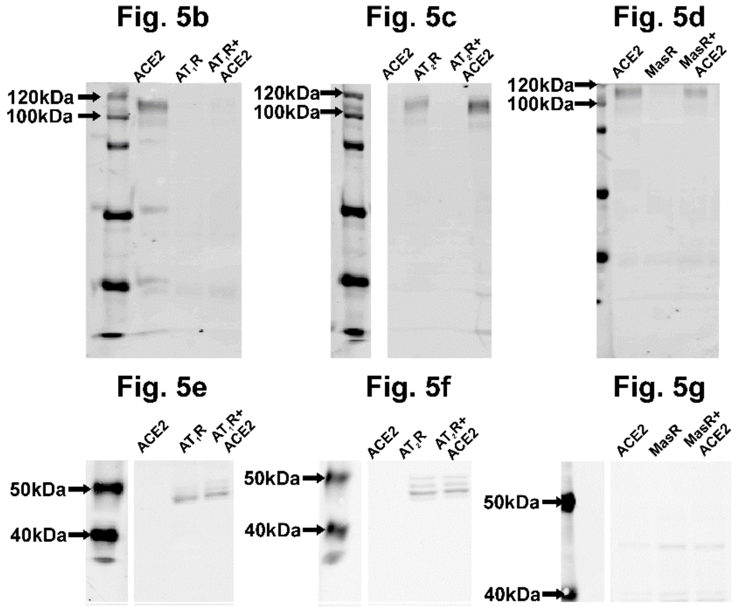

Supplementary Figure S1. Expansion of the image areas in Figure 5 and position of the MW markers corresponding to immunoblots in Figure 5 of the main paper (panels refer to those in Figure 5)

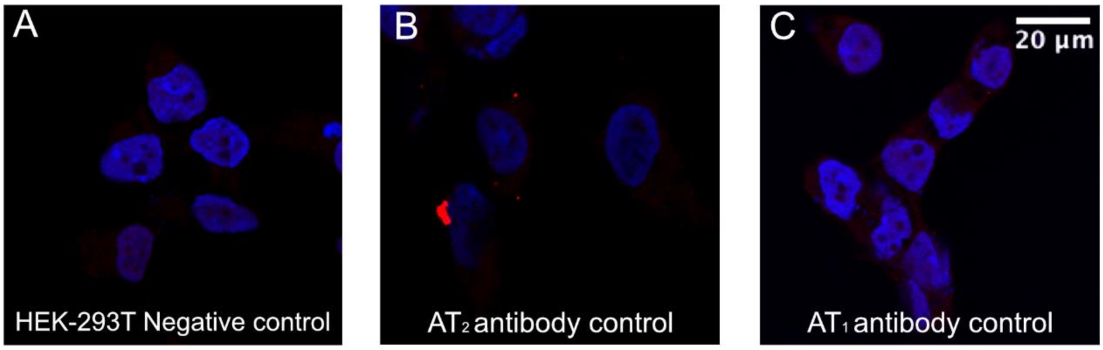

Supplementary Figure S2: Antibody specificity control assays. Immunocytochemistry assays were performed in HEK-293T cells non transfected (**Panel A**), or expressing the human AT_1 (**Panel B**) or the human AT_2 (**Panel C**) receptors (1 µg cDNA each). Cells were incubated with the primary antibody (see Methods for vendor and reference) for AT_1R (A and C) or AT_2R (B) and subsequently incubated with a Cy3 anti-rabbit secondary antibody (red). Images were taken in a Zeiss 880 confocal microscope with 63X-oil objective.
